# Effect of Conventional Physiotherapy on Bladder Control and Quality of Life in Patients With Non-traumatic Postoperative Spinal Cord Injury

**DOI:** 10.7759/cureus.111452

**Published:** 2026-06-24

**Authors:** Sakshi P Kavitake, Suraj Kanase

**Affiliations:** 1 Neuro-Physiotherapy, Krishna College of Physiotherapy, Krishna Vishwa Vidyapeeth (Deemed to be University), Karad, IND

**Keywords:** bladder training, neurogenic bladder, non-traumatic spinal cord injury, pelvic floor muscle training, physiotherapy, quality of life

## Abstract

Introduction

Non-traumatic spinal cord injury (NTSCI) arising from postoperative complications of degenerative spinal disorders represents a clinically significant cause of neurogenic bladder dysfunction and diminished quality of life. Neurogenic lower urinary tract dysfunction is one of the most distressing sequelae of spinal cord involvement, leading to urinary incontinence, incomplete bladder emptying, and recurrent urinary tract infections. Conventional physiotherapy, incorporating pelvic floor muscle training (PFMT), bladder training, core strengthening, and functional mobility exercises, offers a structured, non-pharmacological rehabilitation strategy. However, evidence specifically addressing its role in bladder control and quality of life in this population remains limited. This study investigated the effect of a four-week conventional physiotherapy program on bladder control and quality of life in patients with non-traumatic postoperative spinal cord injury (SCI).

Methods

A single-group pre-post interventional study was conducted at Krishna College of Physiotherapy, Krishna Vishwa Vidyapeeth, Karad. Twenty participants (mean age: 52.40 + 9.87 years; 12 males, 8 females; American Spinal Injury Association (ASIA) Impairment Scale (AIS) grades C and D) diagnosed with non-traumatic postoperative SCI (predominantly lumbar spondylosis with laminectomy/discectomy) were enrolled. Participants underwent a structured four-week conventional physiotherapy program, five sessions per week, each lasting 45-60 minutes. Bladder control was assessed using the validated Neurogenic Bladder Symptom Score (NBSS) and bladder diary parameters; quality of life was evaluated using the validated World Health Organization Quality of Life Brief Version (WHOQOL-BREF) questionnaire. Urodynamic study (UDS), uroflowmetry, and upper urinary tract imaging were not performed in this study. Data were collected at baseline and at four weeks post-intervention and analyzed using a paired t-test.

Results

Following four weeks of conventional physiotherapy, statistically significant improvements were observed in both bladder control and quality of life. The mean NBSS score improved significantly from 35.80 ± 4.62 at baseline to 22.45 ± 3.87 post-intervention (p < 0.001). The mean WHOQOL-BREF total score improved from 52.30 ± 6.14 to 68.75 ± 5.43 (p < 0.001), indicating meaningful gains in the overall quality of life. These findings demonstrated significant improvements in bladder symptoms and quality of life following the intervention; however, causal conclusions regarding treatment effectiveness cannot be established due to the absence of a control group.

Conclusion

A structured four-week conventional physiotherapy program produced significant improvements in neurogenic bladder control and quality of life in patients with non-traumatic postoperative SCI. These findings suggest that conventional physiotherapy may be a beneficial rehabilitation strategy for improving bladder control and quality of life in patients with non-traumatic postoperative SCI. However, controlled studies with larger sample sizes and longer follow-up periods are required to confirm these preliminary findings.

## Introduction

Non-traumatic spinal cord injury (NTSCI) is an increasingly recognized cause of spinal cord injury (SCI) worldwide [1]. It most frequently results from degenerative spinal disorders, spinal stenosis, disc herniation, spinal tumors, vascular malformations, and infective or inflammatory conditions of the spine [2]. Among these, postoperative complications arising from decompressive spinal surgeries, such as laminectomy and discectomy, represent an important subgroup where neurological deficits may develop or persist despite surgical intervention [3].

Spinal cord involvement, irrespective of etiology, invariably leads to disruption of the neural pathways governing lower urinary tract function, resulting in neurogenic lower urinary tract dysfunction (NLUTD) [4]. Neurogenic bladder dysfunction manifests as urinary incontinence, urgency, incomplete bladder emptying, overflow incontinence, and recurrent urinary tract infections, all of which substantially compromise the physical, psychological, and social dimensions of quality of life [5]. The impact of bladder dysfunction extends beyond mere urological inconvenience, often contributing to social isolation, depression, anxiety, reduced activity participation, and caregiver burden [6].

Physiotherapy rehabilitation plays a central role in the comprehensive management of SCI by addressing motor deficits, functional mobility, and secondary complications [7]. In the context of neurogenic bladder dysfunction, conventional physiotherapy interventions, including pelvic floor muscle training (PFMT), bladder diary-guided timed voiding, bladder training, core strengthening exercises, and functional mobility training, have demonstrated potential benefits in improving urinary control and bladder regulation [8]. PFMT, in particular, has been widely used to improve sphincteric control and urethral closure pressure, thereby reducing episodes of urinary incontinence [9]. Bladder training through scheduled voiding aims to progressively improve bladder capacity and reduce urgency by reconditioning bladder reflexes [10].

Despite the growing body of evidence supporting physiotherapy-based bladder rehabilitation in neurological populations, specific evidence regarding its role in patients with NTSCI following spinal surgery remains limited and heterogeneous [11]. Most existing studies have focused on traumatic SCI, multiple sclerosis, or stroke, with relatively few studies specifically addressing postoperative NTSCI populations [12]. Moreover, the combined effect of physiotherapy on both bladder control and broader quality of life outcomes in this population has not been adequately investigated [13].

The quality of life of individuals with NTSCI is influenced not only by neurological deficits and bladder dysfunction but also by pain, physical deconditioning, psychological well-being, and social functioning [14]. A comprehensive rehabilitation approach targeting these multidimensional aspects may therefore be more effective than interventions focused solely on motor recovery [15]. Conventional physiotherapy, by addressing functional mobility, pelvic floor strength, bladder regulation, and physical conditioning simultaneously, offers a holistic rehabilitation strategy for this population [16].

This study, therefore, aims to evaluate the effect of a structured four-week conventional physiotherapy program on bladder control and quality of life in patients with non-traumatic postoperative SCI, using the Neurogenic Bladder Symptom Score (NBSS) and the World Health Organization Quality of Life Brief Version (WHOQOL-BREF) as primary outcome measures.

## Materials and methods

This single-group pre-post interventional study was conducted from December 2025 to April 2026 at Krishna College of Physiotherapy, Krishna Vishwa Vidyapeeth, Karad, over a period of six months. A total of 20 participants diagnosed with non-traumatic postoperative SCI were selected using a purposive sampling method. The Krishna Vishwa Vidyapeeth Institutional Ethics Committee permitted the initiation of the research project (protocol number 280/2025-2026; approval no.: KVV/IEC/10/2025) on November 5, 2025. All participants were recruited following institutional ethics committee approval, and written informed consent was obtained from each participant before enrolment.

Inclusion and exclusion criteria

Participants aged 30 to 75 years with a confirmed diagnosis of NTSCI (partial or complete) following spinal surgery, with neurogenic lower urinary tract dysfunction confirmed by clinical assessment, and with a postoperative status of at least four weeks were included in the study. Participants who were medically stable, able to comprehend and follow instructions, willing to attend regular therapy sessions, and capable of providing informed consent were also included. Participants with traumatic SCI, active urinary tract infection at the time of enrolment, an indwelling urinary catheter that could not be removed, severe cognitive impairment, active pressure ulcers, or any unstable medical condition that could interfere with physiotherapy participation were excluded from the study.

Outcome measures

Two primary outcome measures were used in this study. The Neurogenic Bladder Symptom Score (NBSS) is a validated patient-reported outcome measure specifically designed for neurogenic lower urinary tract dysfunction. It comprises 24 items assessing urinary incontinence, storage and voiding symptoms, and quality of life consequences of bladder dysfunction. Higher NBSS scores indicate greater symptom burden, and a reduction in score reflects clinical improvement in bladder control. The WHOQOL-BREF is a validated 26-item instrument derived from WHOQOL-100, assessing the quality of life across four domains: physical health, psychological well-being, social relationships, and environment. Higher domain scores indicate better quality of life. Both instruments were administered at baseline (pre-intervention) and at the completion of four weeks of physiotherapy (post-intervention). In addition, bladder diary parameters, including mean daily voiding frequency, urgency episodes, and incontinence episodes, were recorded as supplementary voiding outcome measures, providing a multi-parameter assessment of bladder function beyond the NSS alone. It is acknowledged that additional validated instruments, such as the Overactive Bladder Symptom Score (OABSS) and the International Consultation on Incontinence Questionnaire (ICIQ), along with objective measures including uroflowmetry and postvoid residual volume assessment, would have further strengthened the outcome assessment framework. Urodynamic study (UDS), uroflowmetry, and upper urinary tract imaging were not performed in the study. 

Intervention protocol

Table 1 shows that the rehabilitation protocol was implemented over four weeks, with supervised sessions conducted five times per week, each lasting approximately 45-60 minutes. The intervention was progressively structured to target pelvic floor muscle strengthening, bladder regulation, core stability, and functional mobility.

**Table 1:** Four-week structured physiotherapy intervention protocol for neurogenic bladder rehabilitation. ROM: range of motion; LL: lower limb; PF: pelvic floor; PFMT: pelvic floor muscle training

Week	Pelvic floor and bladder training	Exercise and physical rehabilitation	Bladder diary and voiding schedule	Patient education
Week 1	Pelvic floor awareness and identification exercises; supine pelvic floor contractions 3 × 10 reps, 5-sec hold	Diaphragmatic and thoracic breathing exercises; passive and active-assisted lower limb ROM exercises	Bladder diary initiated (voiding frequency, urgency, incontinence); timed voiding commenced at two-hourly intervals	Education on neurogenic bladder mechanisms; hygiene, positioning, and physiotherapy importance
Week 2	Kegel exercises progressed 3 × 15 reps, 8-sec hold/8-sec relax; bladder training with progressive voiding interval extension	Transverse abdominal contractions; pelvic tilting exercises, active-assisted and active LL strengthening (hip flexion, extension, abduction-supine)	Bladder diary continued; progressive voiding interval extension commenced	Sitting balance activities with trunk stabilization initiated
Week 3	Fast-twitch pelvic floor contractions for urge suppression; bladder diary review to guide further interval extension	Progressive core strengthening: pelvic bridging, posterior pelvic tilting; supported sit-to-stand activities; sensory integration and proprioception exercises	Bladder diary review; guided timed voiding interval extension	Standing balance activities with support; Functional transfer training incorporated
Week 4	PFMT at maximum voluntary contraction levels; urge suppression techniques reinforced (PF contraction, distraction, positional strategies)	Functional mobility training: supported gait, obstacle negotiation, stair practice	Bladder strategy integration with functional activity	Home exercise program devised; caregiver education for post-discharge continuation

Statistical analysis

All data were entered into Microsoft Excel (Microsoft Corp., Redmond, WA, USA) and analyzed using GraphPad InStat software (version 3.06, trial version, GraphPad Software, Inc., San Diego, CA, USA). Descriptive statistics, including mean and standard deviation (SD), were calculated for all outcome measures. A paired t-test was used to compare pre-intervention and post-intervention scores for NBSS and WHOQOL-BREF. A p-value of less than 0.05 was considered statistically significant. Effect sizes were calculated to assess the magnitude of treatment effects, and 95% confidence intervals (CIs) were reported for key comparisons.

## Results

A total of 20 participants were enrolled and completed the four-week conventional physiotherapy intervention. No dropouts occurred during the study period. Table 2 presents the demographic and clinical characteristics of the study participants.

**Table 2:** Demographic and clinical characteristics of the study participants AIS: American Spinal Injury Association (ASIA) Impairment Scale; CIC: clean intermittent catheterization; SD: standard deviation

Characteristic	Value
Total participants (n)	20
Age (years), mean ± SD	52.40 ± 9.87
Gender: male, n (%)	12 (60)
Gender: female, n (%)	8 (40)
Level of spinal cord involvement (most common)	Lumbar (L3-L4)
Underlying diagnosis (most common)	Lumbar spondylosis with spinal stenosis
Etiology	Degenerative spinal disorders
Type of surgery (most common)	Laminectomy with discectomy
AIS Grade: C, n (%)	9 (45)
AIS Grade: D, n (%)	11 (55)
Time since onset of neurological dysfunction (weeks), mean ± SD	8.30 ± 3.12
Duration of postoperative status (weeks), mean ± SD	8.30 ± 3.12
Baseline bladder management	Clean intermittent catheterization (CIC) or timed voiding; no indwelling catheter at enrolment

Table 3 presents a comparison of pre-intervention and post-intervention NBSS values. A statistically significant reduction in NBSS scores was observed following the four-week intervention, indicating marked improvement in neurogenic bladder control. The mean NBSS score decreased from 35.80 ± 4.62 at baseline to 22.45 ± 3.87 post-intervention, with a mean improvement of 13.35 ± 3.14 (t = 19.01, p < 0.001).

**Table 3:** Comparison of pre- and post-intervention Neurogenic Bladder Symptom Score (NBSS) Lower scores indicate better bladder control. NBSS: Neurogenic Bladder Symptom Score; SD: standard deviation; QoL: quality of life

Parameter	Pre-intervention mean ± SD	Post-intervention mean ± SD	Mean improvement	t-value	p-value	Significance
NBSS total score	35.80 ± 4.62	22.45 ± 3.87	13.35 ± 3.14	19.01	< 0.001	Highly significant
NBSS incontinence subscale	12.40 ± 2.18	7.65 ± 1.93	4.75 ± 1.52	13.98	< 0.001	Highly significant
NBSS storage/voiding subscale	14.60 ± 2.34	9.30 ± 2.01	5.30 ± 1.64	14.45	< 0.001	Highly significant
NBSS QoL consequence subscale	8.80 ± 1.67	5.50 ± 1.40	3.30 ± 1.18	12.49	< 0.001	Highly significant

Table 4 presents a comparison of pre-intervention and post-intervention WHOQOL-BREF scores. Statistically significant improvements were observed across all four domains and in the total WHOQOL-BREF score following the intervention. The mean total WHOQOL-BREF score improved from 52.30 ± 6.14 at baseline to 68.75 ± 5.43 post-intervention (mean improvement: 16.45 ± 4.32, t = 17.03, p < 0.001). All domain-specific improvements were equally significant, indicating broad gains in physical health, psychological well-being, social relationships, and environmental quality of life.

**Table 4:** Comparison of pre- and post-intervention WHOQOL-BREF scores Higher scores indicate a better quality of life. WHOQOL-BREF: World Health Organization Quality of Life-Brief Version; SD: standard deviation

WHOQOL-BREF domain	Pre-intervention mean ± SD	Post-intervention mean ± SD	Mean improvement	t-value	p-value	Significance
Physical health	48.20 ± 7.32	64.85 ± 6.10	16.65 ± 4.51	16.50	< 0.001	Highly significant
Psychological well-being	50.40 ± 6.85	67.30 ± 5.72	16.90 ± 4.28	17.65	< 0.001	Highly significant
Social relationships	55.10 ± 8.20	70.60 ± 6.44	15.50 ± 4.89	14.18	< 0.001	Highly significant
Environment	54.80 ± 7.64	69.50 ± 6.08	14.70 ± 4.11	15.99	< 0.001	Highly significant
Total WHOQOL-BREF score	52.30 ± 6.14	68.75 ± 5.43	16.45 ± 4.32	17.03	< 0.001	Highly significant

Table 5 presents a comparison of voiding frequency parameters documented through bladder diary assessment before and after the intervention. Significant reductions in the mean daily voiding frequency, urgency episodes per day, and incontinence episodes per day were observed, indicating clinically meaningful improvements in bladder regulation following conventional physiotherapy.

**Table 5:** Comparison of bladder diary parameters before and after intervention SD: standard deviation

Parameter	Pre-intervention mean ± SD	Post-intervention mean ± SD	t-value	p-value	Significance
Mean daily voiding frequency	11.40 ± 2.32	7.85 ± 1.74	7.18	< 0.001	Highly significant
Mean daily urgency episodes	5.60 ± 1.87	2.45 ± 1.23	8.04	< 0.001	Highly significant
Mean daily incontinence episodes	4.30 ± 1.56	1.70 ± 1.08	7.99	< 0.001	Highly significant

## Discussion

This study investigated the effect of a structured four-week conventional physiotherapy program on bladder control and quality of life in patients with non-traumatic postoperative SCI. The findings demonstrated statistically significant improvements in neurogenic bladder control and WHOQOL-BREF domains, suggesting that conventional physiotherapy may contribute positively to rehabilitation outcomes in this population.

NTSCI following postoperative complications disrupts supraspinal and spinal control of the lower urinary tract, producing detrusor overactivity, underactivity, and detrusor-sphincter dyssynergia [17]. The severity of dysfunction depends on lesion level, completeness, and residual neural connectivity [18]. The predominance of incomplete lesions (American Spinal Injury Association (ASIA) Impairment Scale (AIS) grades C and D) in the present cohort likely enhanced treatment responsiveness, as preserved supraspinal input facilitates neuroplastic adaptation [19].

PFMT formed the cornerstone of the intervention. PFMT enhances periurethral muscle strength, improves urethral closure, and reduces detrusor overactivity via afferent inhibitory pathways [20]. Progressive advancement from basic identification contractions to maximal voluntary and fast-twitch recruitment facilitated motor relearning, contributing to significant improvements in NBSS incontinence and storage subscale scores, consistent with previous literature in neurological populations [21].

Bladder training through timed voiding and urge suppression systematically reconditioned lower urinary tract behavior, reducing detrusor overactivity [22]. Significant reductions in voiding frequency, urgency, and incontinence episodes confirm the effectiveness of this approach in patients with post-surgical NTSCI.

Core strengthening and functional mobility training further supported rehabilitation by improving trunk control, pelvic stability, and independence in voiding-related tasks, including self-catheterization [23,24]. The comprehensive approach, therefore, addressed both neurogenic bladder dysfunction and functional impairments, compounding its impact on daily living.

Improvements across all WHOQOL-BREF domains confirm that physiotherapy benefits extended beyond urological function. Bladder dysfunction is known to cause depression, social withdrawal, and loss of autonomy in SCI [25]. Reductions in symptom burden likely alleviated psychological distress and social limitations while motor rehabilitation gains contributed to enhanced self-efficacy and well-being [26-28]. These findings align with existing evidence from multiple sclerosis, stroke, and traumatic SCI populations [29,30], and are notable for addressing the underrepresented group of patients with non-traumatic postoperative SCI.

This study has several important limitations that must be considered when interpreting the findings.

First, the absence of a control group represents the primary methodological limitation. As a single-group pre-post interventional design, causal inferences regarding treatment efficacy cannot be drawn, and comparison of treatment outcomes against a control or comparator group was not possible. This limits the strength of evidence generated and should be addressed in future randomized controlled trials. Second, a significant objective limitation is the absence of a urodynamic evaluation. Comprehensive neurogenic bladder assessment ideally requires UDS, including bladder capacity measurement, detection of detrusor overactivity, detrusor-sphincter dyssynergia, postvoid residual urine volume, voiding efficiency, uroflowmetry parameters, and upper urinary tract imaging. These investigations were not conducted due to resource and facility constraints at the study site. As a result, the physiological basis of the observed symptomatic improvements cannot be fully established, and it remains difficult to determine the physiological significance of the reported improvements with confidence. Symptomatic gains reported through NSS and WHOQOL-BREF cannot be directly attributed to specific physiological changes such as improved detrusor compliance, reduced detrusor overactivity, or enhanced sphincteric function without objective corroboration. Third, the patient population, while clinically relevant, was inadequately characterized in the original report. Detailed neurological characterization, including granular ASIA motor and sensory scores, precise lesion level documentation for all participants, and urodynamic lesion classification, was not uniformly recorded, limiting subgroup analysis by lesion level and completeness. Because neurogenic lower urinary tract dysfunction is highly dependent on lesion level and completeness, these omissions limit interpretation of the results. The revised Table 2 now includes the level of spinal cord involvement, etiology, surgical details, and baseline bladder management to address this concern. Fourth, the study relied exclusively on patient-reported outcome measures and bladder diary parameters. The absence of objective functional assessments, including urodynamics, uroflowmetry, and postvoid residual ultrasound, limits the ability to determine the physiological significance of observed improvements. Future studies must incorporate these objective measures alongside patient-reported outcomes. Fifth, the small sample size (n=20), single-center design, and short four-week follow-up period further limit the generalizability of findings. Long-term follow-up data are needed to assess the durability of treatment efforts. 

## Conclusions

This study demonstrates that a structured four-week conventional physiotherapy program produces significant improvements in neurogenic bladder control and quality of life in patients with non-traumatic postoperative SCI. The combination of PFMT, bladder training with progressive voiding interval extension, core strengthening, and functional mobility exercises resulted in meaningful reductions in urinary incontinence, urgency, and voiding frequency, along with substantial gains in physical, psychological, social, and environmental quality of life. These findings suggest that conventional physiotherapy may be a beneficial rehabilitation strategy for improving bladder control and quality of life in patients with non-traumatic postoperative SCI. Given the absence of a control group, these results should be regarded as preliminary, and further controlled studies are needed before firm clinical recommendations can be made.

However, the limitations of this study, including the single-group design without a control group, the absence of urodynamic evaluation, inadequate neurological characterization, and short follow-up, must be acknowledged. The physiological significance of the observed improvements cannot be fully established without objective functional assessment, including UDS, uroflowmetry, and postvoid residual measurement. Future randomized controlled trials with larger sample sizes, extended long-term follow-up, complete neurological profiling, and comprehensive urodynamic evaluation are strongly recommended. Future studies should also incorporate a broader battery of validated instruments, including the OABSS, ICIQ, and objective urodynamic parameters, to provide a comprehensive multidimensional evaluation of neurogenic bladder function and to establish definitive clinical guidelines for this population.
